# Global Transcriptome Profiling of the Pine Shoot Beetle, *Tomicus yunnanensis* (Coleoptera: Scolytinae)

**DOI:** 10.1371/journal.pone.0032291

**Published:** 2012-02-23

**Authors:** Jia-Ying Zhu, Ning Zhao, Bin Yang

**Affiliations:** 1 Key Laboratory of Forest Disaster Warning and Control of Yunnan Province, College of Forestry, Southwest Forestry University, Kunming, China; 2 College of Life Sciences, Southwest Forestry University, Kunming, China; Kyushu Institute of Technology, Japan

## Abstract

**Background:**

The pine shoot beetle *Tomicus yunnanensis* (Coleoptera: Scolytinae) is an economically important pest of *Pinus yunnanensis* in southwestern China. Developed resistance to insecticides due to chemical pesticides being used for a long time is a factor involved in its serious damage, which poses a challenge for management. In addition, highly efficient adaptation to divergent environmental ecologies results in this pest posing great potential threat to pine forests. However, the molecular mechanisms remain unknown as only limited nucleotide sequence data for this species is available.

**Methodology/Principal Findings:**

In this study, we applied next generation sequencing (Illumina sequencing) to sequence the adult transcriptome of *T. yunnanensis*. A total of 51,822,230 reads were obtained. They were assembled into 140,702 scaffolds, and 60,031 unigenes. The unigenes were further functionally annotated with gene descriptions, Gene Ontology (GO), Clusters of Orthologous Groups (COG), and Kyoto Encyclopedia of Genes and Genome (KEGG). In total, 80,932 unigenes were classified into GO, 13,599 unigenes were assigned to COG, and 33,875 unigenes were found in KO categories. A biochemical pathway database containing 219 predicted pathways was also created based on the annotations. In depth analysis of the data revealed a large number of genes related to insecticides resistance and heat shock protein genes associated with environmental stress.

**Conclusions/Significance:**

The results facilitate the investigations of molecular resistance mechanisms to insecticides and environmental stress. This study lays the foundation for future functional genomics studies of important biological questions of this pest.

## Introduction

The bark beetle genus *Tomicus* (Coleoptera: Curculionidae: Scolytinae) comprises seven described species [1]. Among them, *T. piniperda* and *T. minor* have a Palearctic distribution from Europe to Japan and China and repeatedly introduced to North America, *T. destruens* is found only around the Mediterranean Basin, while *T. yunnanensis*, *T. brevipilosus*, *T. pilifer* and *T. puellus* are restricted to southwestern, eastern, and central China [2]. *Tomicus* damages various pine species mainly due to its shoot feeding behavior and trunk stem attacking, which is among the most damaging pests of pine forests in the outbreak countries. Especially, some species of *Tomicus* have evolved towards different ecological strategies of host use, wider range distribution, and high levels of resistance to major classes of insecticides owing to biological and genetic factors. They are as exotic pests frequently invaded into new areas. *Tomicus* imposes a potentially serious damage upon pine forest.

Having a globally economical importance and ecological threat, it has been studied well from general biology and ecological viewpoints [3]–[5]. But it has remained poorly investigated at the molecular level. Only few studies have been developed, leading to elucidate their population genetic and species evolution [2], [6]. Up to September 26, 2011, there are only 719 nucleotide sequences deposited on National Center for Biotechnology Information (NCBI) for *Tomicus*. Due to insufficient genetic background, the molecular mechanisms of *Tomicus* in forest ecological systems are poorly understood.

The next generation sequencing (high-throughput deep sequencing) technology recently enables efficient approach on large-scale and genome-wide for gene discovery and expression profiling, and studies in functional, comparative and evolutionary genomics in non-model organisms with little or no previous genomic information exists [7], [8]. In the past few years, several studies based on this technology have, indeed, allowed the efficient, massive and successful molecular mechanisms investigation of some insect species lacking genome information, such as *Bemisia tabaci*, *Nilaparvata lugens*, and *Trialeurodes vaporariorum* [9]–[11].

*T. yunnanensis* is a newly described pine shoot beetle only found in Yunnan, Sichuan, and Guizhou Provinces in southwestern China, and has affected 200,000 ha. of *Pinus yunnanensis* forests over the past 30 years [12]. Control of this pest until now depended largely on chemical insecticides. It has evolved high level of resistance to insecticides. This might be due to the increasing of metabolic capability of detoxificative systems and/or reducing target site sensitivity. In addition, this pest has been found distributed in divergent environmental ecologies. The distributional areas of *T. yunnanensis* are with high mounts and deep valley as geographical barriers, and its ecological environments are in large difference. It suggested that *T. yunnanensis* adapted efficiently to environmental stress for surviving in diversifiable ecologies during the past years. Even resistance of *T. yunnanensis* to insecticides and severe environmental stress is an ongoing challenge for pest management, there is no information available for uncovering the molecular mechanisms under these.

In the present work, we characterized the first global transcriptome using Illumina technology of that *Tomicus* species *T. yunnanensis*. A systematic bioinformatics strategy was engaged to functional annotation of the transcriptome data. Additionally, important homologues of genes involved in insecticide resistance and heat shock protein (HSP) genes associated with environmental stress were identified. This study dramatically provides a foundation and increases the significant promise for further functional genomics studies of *T. yunnanensis* and other *Tomicus* species.

## Materials and Methods

### Ethics statement

Regarding the field study, no specific permits were required. The location is not privately-owned or protected in any way. The field studies did not involve endangered or protected species.

### Insects

Adult beetles identified based on morphological characters [1] as *T. yunnanensis* by their trunk attacking phase on *P. yunnanensis* were collected from Qujing city, Yunnan province, China. The samples were frozen at −80°C until use.

### cDNA library and Illumina sequencing

Total RNA was extracted from each 20 female and male adult beetles using TRIzol® Reagent (Invitrogen) according to the manufacturer's instructions. RNA quality and yield were assessed by 2100 Bioanalyzer (Agilent Technologies) with a minimum RNA integrated number value of 8. The cDNA library was prepared using Illumina's kit following manufacturer's recommendations. Briefly, messenger RNA was isolated from 20 µg total RNA (pooled RNA of female and male adults) using oligo(dT) magnetic beads and fragmented into short sequences using divalent cations under elevated temperature. First and second strand cDNA were synthesized from cleaved RNA fragments. After the end repair and ligation of adaptors, the products were cleaned up with a QIAquick PCR Purification Kit to create the final cDNA library. The library was sequenced on the Illumina sequencing platform (GAII) to obtain short sequences from both ends.

### Bioinformatic analysis

The raw reads from the images and quality value calculation were performed by the Illumina data processing pipeline (version 1.6). Before the assembly, the raw reads were cleaned by adaptor sequences, empty reads and low quality sequences (reads with unknown sequences ‘N’) to obtain the high-quality clean reads. Raw reads were then assembled into sequence contigs, scaffolds, and unigenes using SOAPdenovo software [13], and clustered using TGI Clustering tools [14]. All Illumina assembled unigenes were searched against nr database in NCBI, Swiss-Prot, Kyoto Encyclopedia of Genes and Genome (KEGG), and Cluster of Orthologous Groups (COG) with the BLASTX algorithm. The E-value cut-off was set at 10^−5^. Genes were tentatively identified according to the best hits against known sequences. Blast2GO [15] was used to predict the functions of the sequences, assign Gene Ontology (GO) terms, and predict the metabolic pathways in COG and KEGG databases. Amino acid sequence comparisons were conducted with Clustal X (v1.83) program [16]. Phylogenetic tree was constructed by MEGA 5.0 package [17] using the neighbour-joining method with the Poisson correction model after sequence alignment performed by Clustalx. Bootstrap analysis of 1,000 replication trees was performed in order to evaluate the branch strength of each tree.

### Data availability

The Illumina reads of *T. yunnanensis* have been submitted to NCBI Short Read Archive under the accession number of SRA047283.

## Results and Discussion

### Sequencing and *de novo* assembly

**Figure 1:**
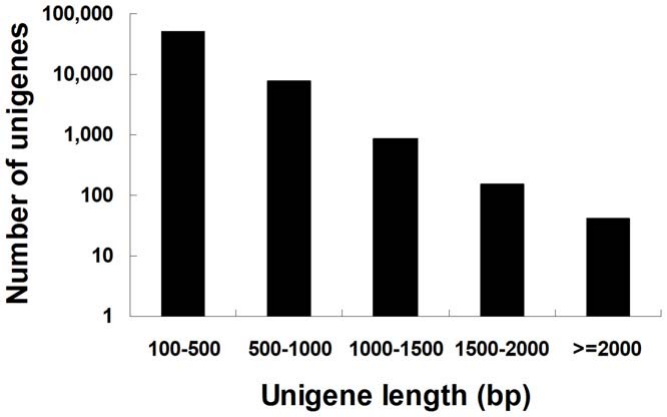
Length distribution of unigenes. The number of y-axis has been transfer into logarithmic scale.

Illumina sequencing resulted in 51,822,230 raw reads, corresponding to an accumulated length of 4,664,000,700 bp (Table 1). The average raw reads length is 90 bp, which is consistent with the Illumina sequencing capacity. Using SOAPdenovo software, the raw reads were assembled into contigs after adaptor sequences, empty reads and low quality sequences filtered out. The raw reads were assembled into 642,521 contigs with a mean length of 117 bp. The range length of contigs is from 50 to 2953 bp. Although the majority of the contigs are between 50 to 400 bp, we obtained 9,331 contigs which were greater than 400 bp in length. The size distribution of these contigs is shown in Figure S1. Using paired end-joining and gap-filling, these contigs were further assembled into 140,702 scaffolds with a mean length of 233 bp, and range length of 100–2953 bp. 2,293 scaffolds were longer than 800 bp (Figure S2). Using TGI software, scaffold sequences were assembled into clusters. We obtained 60,031 unigenes with a mean length of 355 bp. The lengths of the 8,953 and 1,055 unigenes were ≥500 bp and 1000 bp, respectively, revealing that 85% of them fell between 100 and 500 bp in length (Figure 1). The result demonstrated that the scaffold and unigene length distribution followed the contig length distribution closely, with the majority being shorter sequences with relatively little redundancy, which was in a similar to other insect transcriptome projects using this technology [9]–[11], [18], [19]. The majority of scaffolds and unigenes after assembly were still less than 500 bp, which might be due to the short length sequencing capacity of Illumina sequencing and/or the low coverage of the transcriptome represented in this dataset [20]. The assembled abundant sequence data provided a rich source of information for further investigation, thus allowing for rapid characterization of a large portion of the transcriptome and better reference of interesting genes.

**Table 1:** Sequence statistics of the Illumina sequencing assembly.

	Reads	Contigs	scaffolds	Unigenes
Number of sequences	51,822,230	642,521	140,702	60,031
Mean length (bp)	90	117	233	355
Total length (bp)	4,664,000,700	74,951,788	32,749,102	21,338,135

### Annotation of predicted proteins

**Figure 2:**
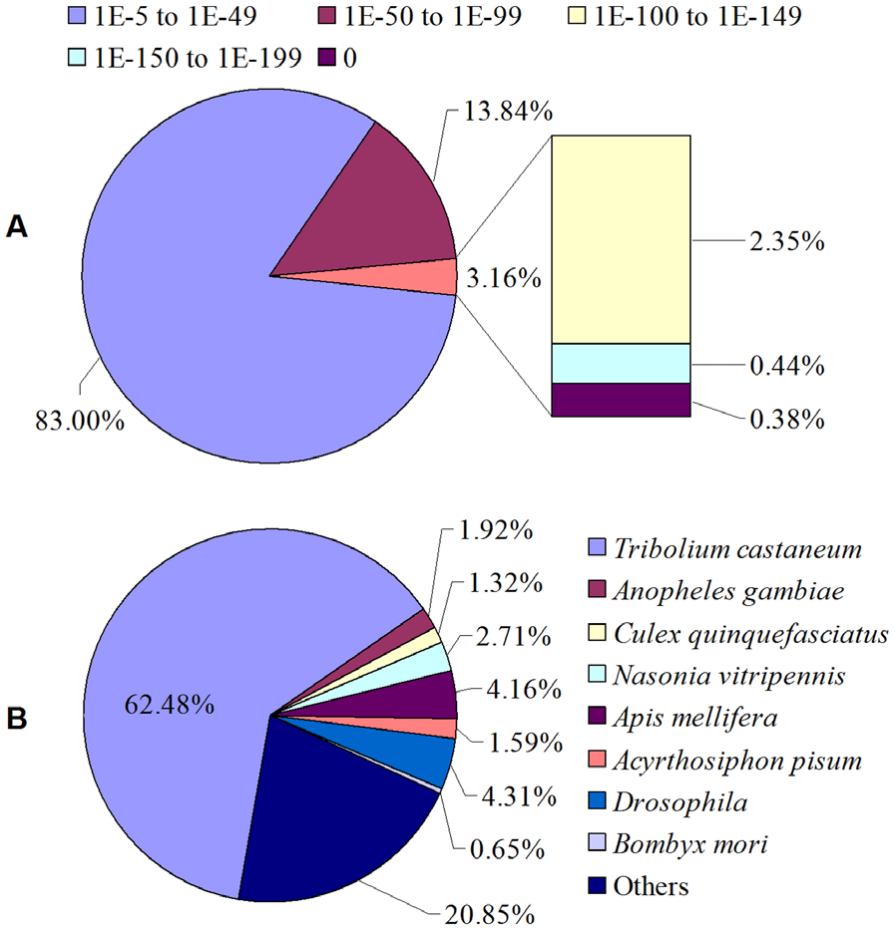
Characteristics of homology search of Illumina sequences against the nr database. (A) E-value distribution of BLAST hits for each unique sequence with a cut-off E-value of 1.0E-5. (B) Species distribution of the BLASTX results. We used the first hit of each sequence for analysis.

The assembled unigenes were used as a query for Blastx searches in the NCBI nr protein database with a cut-off E-value of 10^−5^. The search produced 34,702 hits, which comprised 57.81% of all the unigenes (Table S1). A large proportion of them (about 40%) apparently have no significant match in any of the existing databases, indicating many of them may contain novel sequences and a high number of Coleoptera or species-specific transcripts or transcript parts (orphan UTRs). This is expected, as there is very little sequence information from closely related species. The E-value distribution of the top hits in the nr database showed that 17% of the mapped sequences have strong homology (smaller than 1.0E^−49^), whereas 83% of the homolog sequences ranged between 1.0E^−5^ to 1.0E^−49^ (Figure 2A). The species distribution of the best match result for each sequence showed that the *T. yunnanensis* sequences have 62.48% matches with sequences from the Coleoptera species (*Tribolium castaneum*), while very low proportion (<5%) of them have matches to other insects (Figure 2B). It demonstrated that *T. yunnanensis* have near evolution distance with *T. castaneum*.

### GO assignments

**Figure 3:**
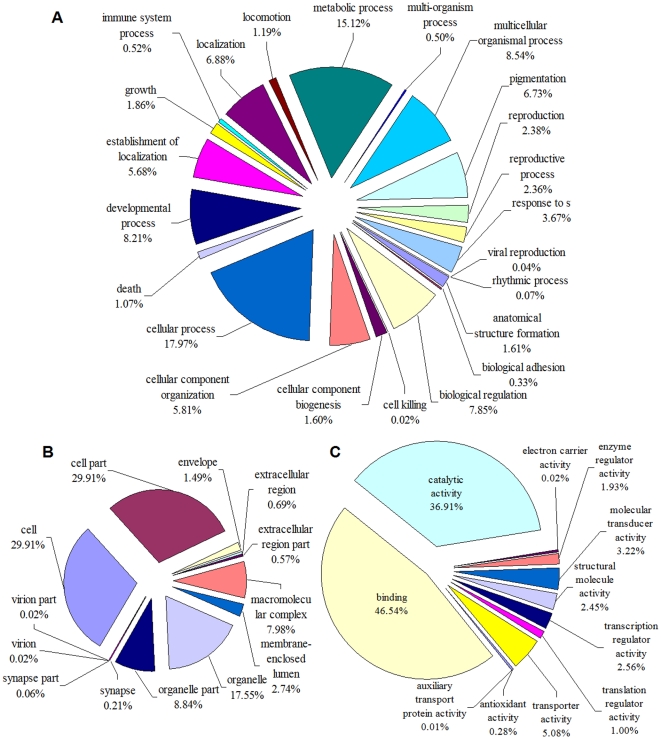
Distribution of second level GO of *Tomicus yunnanensis* transcriptome. (A) biological process, (B) cellular component and (C) molecular function. The percentage of a specific category of genes in that main category is shown.

Gene ontology is widely used to standardize representation of genes across species and provide a controlled vocabulary of terms for describing gene products [21]. In total, 80,932 unigenes were assigned for GO terms based on BlastX matches with sequences whose function is previously known (Figure 3, Table S2). These GO terms were summarized into the 3 main GO categories (biological process, cellular component, and molecular function) according to the standard GO terms and 47 sub-categories. Compared to the GO annotations of *Drosophila melanogaster* genome [22], our sequence data do not contain any notable biases towards particular categories of genes. Biological process made up the majority of the GO annotations (38,578, 47.67% of the total), followed by cellular component (26,119, 32.27%), and molecular function (16,235, 20.06%). Among biological process category, cellular process (17.97%), and metabolic process (15.12%) were the most dominant subcategories, reflecting that the analyzed tissues were undergoing rapid growth and extensive metabolic activities. The following subcategories were multicellular organismal process (8.54%), developmental process (8.21%), biological regulation (7.85%), localization (6.88%), and cellular component organization (5.81%). The biological process illustrated all of the major cellular processes from transport and cellular organization to transcription, translation, and metabolism. Under the category of cellular component, cell (29.91%), cell part (29.91%), and organelle (17.75%) were among the most highly represented subcategories. Most of the unigenes annotated with a cellular component are localized to plastids or mitochondria. The molecular function category was mainly comprised of proteins involved in binding (46.54%), predominantly heat shock proteins (Hsp), and catalytic activities (36.91%) including hydrolases, kinases, and transferases, allowing for the identification of genes involved in the secondary metabolite synthesis pathways. Similar observations for metabolic processes were reported in transcriptomic studies of other insects [18]. These GO annotations represent a general gene expression profile signature for *T. yunnanensis* adults, which demonstrates that the expressed genes in this species encode diverse structural, regulatory and stress proteins.

### COG classification

**Figure 4:**
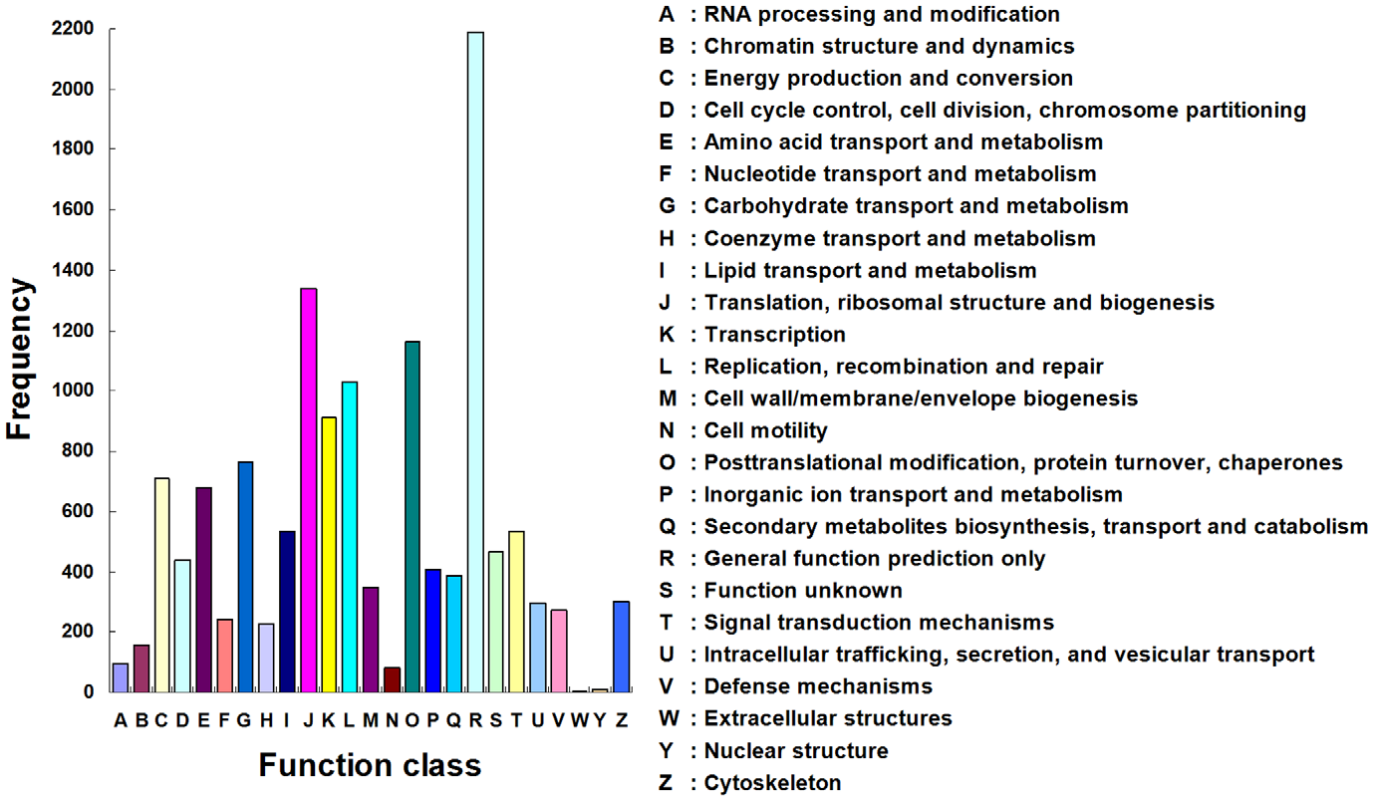
COG function classification of the *Tomicus yunnanensis* transcriptome. All putative proteins were aligned to the COG database and can be classified functionally into at least 25 molecular families.

A total of 13,599 unigenes were assigned to the appropriate COG clusters (Figure 4). These COG classifications were grouped into 25 function categories that correspond to the categories observed in GO analysis. The cluster for general function prediction only (16.10%) represents the largest group. The other three largest categories include: (1) translation, ribosomal structure and biogenesis (9.85%), (2) posttranslational modification, protein turnover, chaperones (8.54%), and (3) replication, recombination and repair (7.54%). The category of secondary metabolites biosynthesis, transport and catabolism was highlighted with 2.86%, because of the importance of secondary metabolites to the insecticides in insects. The most abundant sequences in this category are cytochrome P450 monooxygenases. To some extent, the COG classifications shed light on specific responses and functions involved in the molecular processes of *T. yunnanensis*.

### KEGG analysis

To identify the biological pathways that are active in *T. yunnanensis*, we mapped the 34,702 annotated sequences to the referential canonical pathways in KEGG. A total of 16,727 unigenes were assigned to 219 KEGG pathways. All the pathways are summarized in Table S3. The top 10 pathways are metabolic pathways (3339 members), spliceosome (665 members), huntington's disease (663 members), pathways in cancer (586 members), lysosome (527 members), purine metabolism (509), regulation of actin cytoskeleton (472 members), alzheimer's disease (471 members), ubiquitin mediated proteolysis (467 members), and focal adhesion (466 members). These annotations provide a valuable resource for investigating specific processes, functions and pathways in *T. yunnanensis* research.

### Putative insecticides resistance related genes

#### Cytochrome P450 (P450)

Because of the genetic diversity, broad substrate specificity, and catalytic versatility, P450s can mediate resistance to all classes of insecticides [23]. Approximately a total of 146 P450 related unigenes were identified in the transcriptome. Even 23 *T. yunnanensis* P450 sequences were identified in the dataset as length longer than 600 bp (Table 2), and the majority of them were as short fragments listed in Table S4. In general, insect genomes harbor ∼100 different P450s, which can be divided into four clades (CYP2, CYP3, CYP4 and mito) [24], [25]. The number of unigenes in the *T. yunnanensis* transcriptome belongs to the range of P450s identified in other insect species, while the accurate gene number still remains to be identified by gene cloning based on the fragments obtained here. Of the 23 unigenes which contained longer length, it was possible to differentiate them into four clades by phylogenetic analysis. The majority of these P450s belonged to the CYP3 and CYP4 clades compared with CYP2 and mito clades, which is in agreement with *Tribolium castaneum* and other insect systems [25], [26]. Because some functionality of P450s can be assigned to each of the four clades based on known functionalities characterized in other insects, we can select some interesting candidates for a further investigation according to the phylogenetic results. Until recently, the increased production of P450s in resistant insects has shown to occur almost exclusively through up regulation via changes in *cis*-or *trans*-acting regulatory loci, but gene duplication or amplification of P450s has now been implicated in the resistance of four insect species [27]. From phylogeny, gene duplication events specific to *T. yunnanensis* are apparent, with the best example being the two CYP3-type sequences Unigene58724 and Unigene58733, and two CYP4-type sequences Unigene59997 and Unigene8705, if they were paired in the phylogeny with bootstrap support greater than 70%. However, the relevance of duplication resistance of *T. yunnanensis* requires a further investigation.

**Table 2:** Putatively identified P450 genes in *Tomicus yunnanensis*.

Unigene ID	Putative assignment	Length (bp)	First hit	Identity (%)	E_value	Blast annotation/Organism
Unigene12883	CYP3	612	XP_975568	44	2.00E-28	PREDICTED: similar to cytochrome P450 [*Tribolium castaneum*]
Unigene15066	CYP4	819	EFA01330	58	9.00E-59	cytochrome P450-like protein [*Tribolium castaneum*]
Unigene1549	CYP4	679	AAT38513	42	8.00E-43	ubiquitous cytochrome P450 [*Phyllopertha diversa*]
Unigene1697	CYP3	902	EFA04564	39	5.00E-56	cytochrome P450 347A1 [*Tribolium castaneum*]
Unigene21111	CYP3	771	XP_972794	56	9.00E-34	PREDICTED: similar to Probable cytochrome P450 9f2 (CYPIXF2) [*Tribolium castaneum*]
Unigene22371	CYP4	694	XP_001814854	45	1.00E-45	PREDICTED: similar to cytochrome P450 [*Tribolium castaneum*]
Unigene23650	mito	645	XP_974252	60	7.00E-65	PREDICTED: similar to CYP302a1 [*Tribolium castaneum*]
Unigene23993	CYP2	1092	NP_503303	38	2.00E-57	CYtochrome P450 family member (cyp-33C11) [*Caenorhabditis elegans*]
Unigene24457	CYP3	1192	EEZ99338	62	2.00E-63	cytochrome P450 6BQ13 [*Tribolium castaneum*]
Unigene24875	CYP2	1173	XP_968477	53	1.00E-98	PREDICTED: similar to cytochrome P450, partial [*Tribolium castaneum*]
Unigene551	mito	640	NP_001123894	57	2.00E-54	cytochrome P450 CYP314A1 [*Tribolium castaneum*]
Unigene58533	CYP4	603	ABF06546	67	2.00E-44	CYP4BD1 [*Ips paraconfusus*]
Unigene58724	CYP3	627	NP_001164248	50	5.00E-50	cytochrome P450 9Z4 [*Tribolium castaneum*]
Unigene58733	CYP3	628	NP_001164248	47	4.00E-51	cytochrome P450 9Z4 [*Tribolium castaneum*]
Unigene59248	CYP4	720	ABF06544	46	5.00E-59	CYP4AY1 [*Ips paraconfusus*]
Unigene59398	CYP4	760	XP_973153	45	1.00E-44	PREDICTED: similar to cytochrome P450 [*Tribolium castaneum*]
Unigene59490	CYP4	788	XP_001602395	36	7.00E-41	PREDICTED: similar to cytochrome P450 [*Nasonia vitripennis*]
Unigene59572	CYP3	818	EFA02819	45	6.00E-67	cytochrome P450 6BQ5 [*Tribolium castaneum*]
Unigene59684	CYP3	871	NP_496108	42	4.00E-47	CYtochrome P450 family member (cyp-13A1) [*Caenorhabditis elegans*]
Unigene59753	CYP2	916	XP_969587	69	1.00E-115	cytochrome P450 307A1 [*Tribolium castaneum*]
Unigene59997	CYP4	1443	EEZ99364	45	1.00E-100	cytochrome P450-like protein [*Tribolium castaneum*]
Unigene8212	CYP4	650	ABF06550	60	6.00E-71	CYP4BG1 [*Ips paraconfusus*]
Unigene8705	CYP4	686	EEZ99364	32	4.00E-32	cytochrome P450-like protein [*Tribolium castaneum*]

**Fragments less than 600 bp listed in Table S4**.

#### Glutathione S-transferase (GST)

GSTs catalyse the glutathione conjugation reaction with reduced glutathione (GSH) to convert them, resulting in less toxic water-soluble products that can eventually be excreted, which are widespread in both prokaryotes and eukaryotes [28]. The increased expression and activity of GSTs, and amplification of the structural GST genes has been documented as a mechanism of insect resistance [27]. Seventeen putative GST unigenes were identified in *T. yunnanensis* transcriptome (Table 3). Based on the closest BLAST hits in the NCBI nr database and, when possible, through applying a phylogenetic analysis 14, 2, and 1 unnigenes were assigned to the Epsilon, Sigma, and Delta classes, respectively. In insects, there are two ubiquitously distributed distantly related groups of GSTs, classified according to their location within the cell: microsomal and cytosolic [29]. The microsomal class contains few gene duplicates, while the cytosolic class contains highly diverse larger gene family divided into six major subclasses: Delta, Epsilon, Sigma, Omega, Theta, and Zeta [30]. In regard to microsomal class, it may await discovery due to its absence from the current transcriptomic dataset, but this group has not been implicated in the metabolism of insecticides [29]. In microsomal class, only three subclasses have been identified from the current database, which is similar to some insect species that do not have six subclasses like *Drosophila melanogaster* [31]. For instance, only Sigma, Delta, and Theta type GSTs were found in *Nasonia vitripennis*, and Epsilon, Sigma, and Omega type GSTs were found in *T. castaneum* based on genomic analysis [30]. Although the Delta subclasse is absent in *T. castaneum*, one unnigene assigned to Delta subclasse was identified in *T. yunnanensis*. In addition, the majority of *T. yunnanensis* GSTs were assigned to Epsilon subclasse, which is in accordance with studied insect GSTs with extensive cases of gene expansions in Epsilon and Delta types [32].

**Table 3:** Putatively identified GST genes in *Tomicus yunnanensis*.

Unigene ID	Putative assignment	Length (bp)	First hit	Identity (%)	E_value	Blast annotation/Organism
Unigene22819	Epsilon	521	XP_971136	49	2.00E-25	PREDICTED: similar to Glutathione S transferase E8 CG17533-PA [*Tribolium castaneum*]
Unigene23097	Epsilon	542	ACU09495	41	9.00E-23	glutathione S-transferase 16 [*Helicoverpa armigera*]
Unigene25020	Sigma	693	XP_002633726	35	2.00E-16	C. briggsae CBR-GST-3 protein [*Caenorhabditis briggsae*]
Unigene25058	Epsilon	715	XP_966966	41	1.00E-37	PREDICTED: similar to Glutathione S transferase E6 CG17530-PA [*Tribolium castaneum*]
Unigene28476	Epsilon	209	XP_971389	65	4.00E-15	PREDICTED: similar to Glutathione S transferase E5 CG17527-PA [*Tribolium castaneum*]
Unigene31256	Epsilon	218	XP_966787	42	7.00E-10	PREDICTED: similar to Glutathione S transferase E7 CG17531-PA [*Tribolium castaneum*]
Unigene34958	Epsilon	230	XP_971268	37	2.00E-07	PREDICTED: similar to Glutathione S transferase E7 CG17531-PA [*Tribolium castaneum*]
Unigene35169	Epsilon	231	XP_971449	54	2.00E-11	PREDICTED: similar to Glutathione S transferase E7 CG17531-PA [*Tribolium castaneum*]
Unigene37693	Sigma	241	NP_001165920	52	6.00E-14	glutathione S-transferase S3 [*Nasonia vitripennis*]
Unigene40017	Epsilon	252	NP_611325	56	3.00E-19	glutathione S transferase E3 [*Drosophila melanogaster*]
Unigene44809	Epsilon	279	ADD19697	62	2.00E-27	glutathione S-transferase [*Glossina morsitans morsitans*]
Unigene44817	Epsilon	279	ADD19697	61	1.00E-27	glutathione S-transferase [*Glossina morsitans morsitans*]
Unigene49693	Epsilon	323	XP_967234	43	3.00E-19	PREDICTED: similar to glutathione S-transferase 6A [*Tribolium castaneum*]
Unigene50873	Delta	339	XP_974204	76	4.00E-45	PREDICTED: similar to GST [*Tribolium castaneum*]
Unigene54951	Epsilon	415	BAE80117	45	4.00E-26	glutathione S-transferase [*Plutella xylostella*]
Unigene5052	Epsilon	443	XP_971136	45	2.00E-31	PREDICTED: similar to Glutathione S transferase E8 CG17533-PA [*Tribolium castaneum*]
Unigene8150	Epsilon	416	NP_611328	44	2.00E-14	glutathione S transferase E6 [*Drosophila melanogaster*]

#### Other candidates

In addition to the detailed analyses above, further unigenes were identified with a high sequence similarity to important genes related to insecticide metabolism and targets. As shown in Table 4, a number of unigenes annotated as enzymes related to insecticide metabolic resistance, such as carboxylesterase, and superoxide dismutase; and insecticide targets, such as acetyl-CoA carboxylase, acetylcholinesterase, and γ-aminobutyric acid (GABA) receptor, were present in *T. yunnanensis* transcriptome. Although most of these unigenes are not full length, they will nevertheless facilitate a further characterisation of these targets by RACE to retrieve the full length cDNAs. The abundance of these transcripts demonstrates the quality of our sequencing data. It provided new leads for functional studies of dissecting the potential insecticide resistance role each these genes plays.

**Table 4:** Putative genes of interest related to insecticide resistance.

Gene name	Uumber of unigenes had a hit with nr database
Cytochrome P450	146
Glutathione S-transferase	17
Carboxylesterase	128
Superoxide dismutase	20
Acetyl-CoA carboxylase	25
Acetylcholinesterase	6
γ-aminobutyric acid (GABA) receptor	8
Nicotinic acetylcholine receptor	17
Sodium channel	5
Chloride channel	63
Ryanodine receptor	36

### Detection of HSP genes

#### Heat shock protein 10 (HSP10)

**Figure 5:**
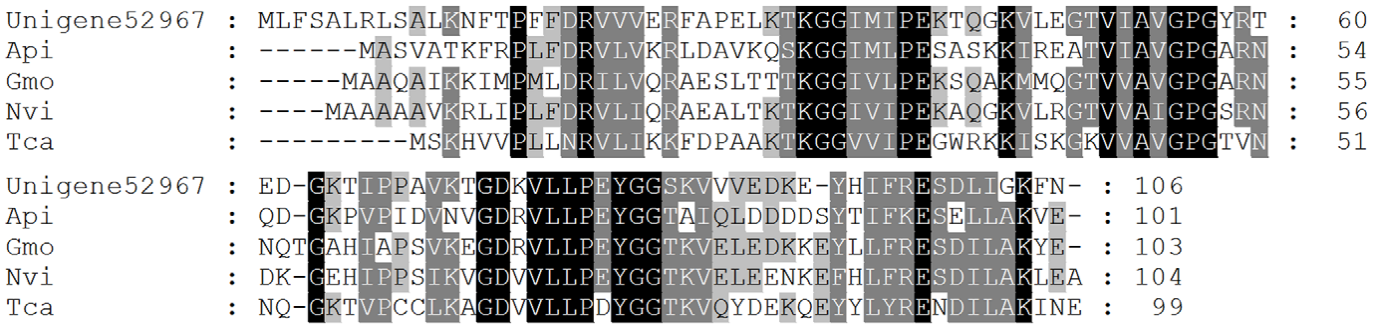
Alignment of the predicted amino acid sequences of *Tomicus yunnanensis* HSP10s with that of other insects. Identical residues are shaded black, conserved substitutions are shaded grey. Dash (–) indicates insertion or deletion. Api, *Acyrthosiphon pisum* (NP_001119666); Gmo, *Glossina morsitans morsitans* (ADD19718); Nvi, *Nasonia vitripennis* (XP_001599992); Tca, *Tribolium castaneum* (XP_969732).

HSPs are highly conserved molecules that play vital roles in all cells. Among them, HSP10 is a near 10 kDa, highly conserved protein. In eukaryotes, HSP10, originally identified as a mitochondrial chaperone, now is also known to be present in other places such as cytosol, cell surface, and extracellular space [33]. Here, six unigenes were identified putatively encoded HSP10 (Table S5). Two of them (Unigene4430 and Unigene52967) appeared to be complete. Amino acid alignment revealed a low homology of these four HSP10s to HSP10s from the *Acyrthosiphon pisum* (46%), *Glossina morsitans morsitans* (49%), *Nasonia vitripennis* (51%), and *Tribolium castaneum* (41%) (Figure 5). HSP10 is known as a co-chaperone for heat shock protein 60 (HSP60), and exerts immunosuppressive activity in mammals [34]. To our best knowledge, HSP10 in insects has not been structurally and functionally studied in detail.

#### Small heat shock protein (sHSP)

**Figure 6:**
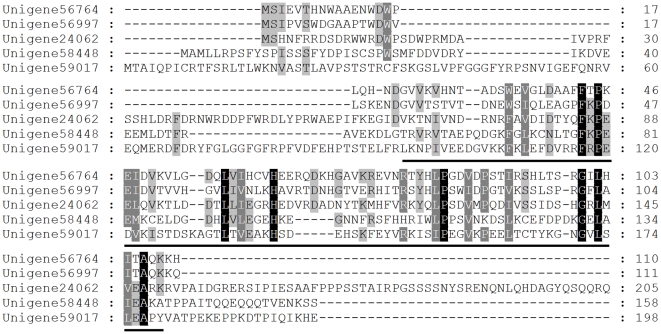
Alignment of the predicted amino acid sequences of *Tomicus yunnanensis* sHSPs. The conserved α-crystallin domain is underlined. Identical residues are shaded black, conserved substitutions are shaded grey. Dash (–) indicates insertion or deletion.

sHSP are a family of molecular chaperones, with molecular mass ranging from 12 kDa to 43 kDa, usually below 30 kDa, which is involved in cellular defense under environmental stress conditions [35], [36]. Up to data, knowledgment of insect sHsps is much less than of sHsps in plants and vertebrates. Twenty three unigenes were found to be with similarities to sHSP (Table S5). Among of them, five (Unigene24062, Unigene56764, and Unigene56997, Unigene58448 and Unigene59017) appeared to be complete. These five sHSPs showed variable sequence identity, ranged from 29% to 82%, to the first hits under blast search, suggesting that sHSPs are diverse. An alignment of the predicted proteins deduced from the complete unigenes is shown in Figure 6. Interestingly, the alignment indicated that they were with very low similarity between each other, revealing that they do not display a highly conservation. Previous studies have demonstrated that sHSPs are abundant and ubiquitous in almost all organisms with different numbers, from bacteria to algae single celled and even to the higher organisms including human [37]. Representative sequences of the many demonstrated sHSPs available share a conserved sequence of approximately 90 amino acid residues, termed α-crystallin domain responsible for dimer formation and form large multimeric complexes that are known to be crucial to their chaperone activities [38]–[40]. There is α-crystalling domain in the resided C-terminus of *T. yunnanensis* predicted sHSPs. The N-terminal coding sequences and N-terminal ends of sHSPs are more variable than C-terminal with the α-crystalling domain in different species [41]. As expected, the deduced amino acid sequence of *T. yunnanensis* HSP20s showed high conservation in α-crystalling domain, and high divergence in N-terminal region and variable N-terminal end. However, there are still some conserved amino acid residues in N-terminal.

#### Heat shock protein 40 (HSP40)

**Figure 7:**
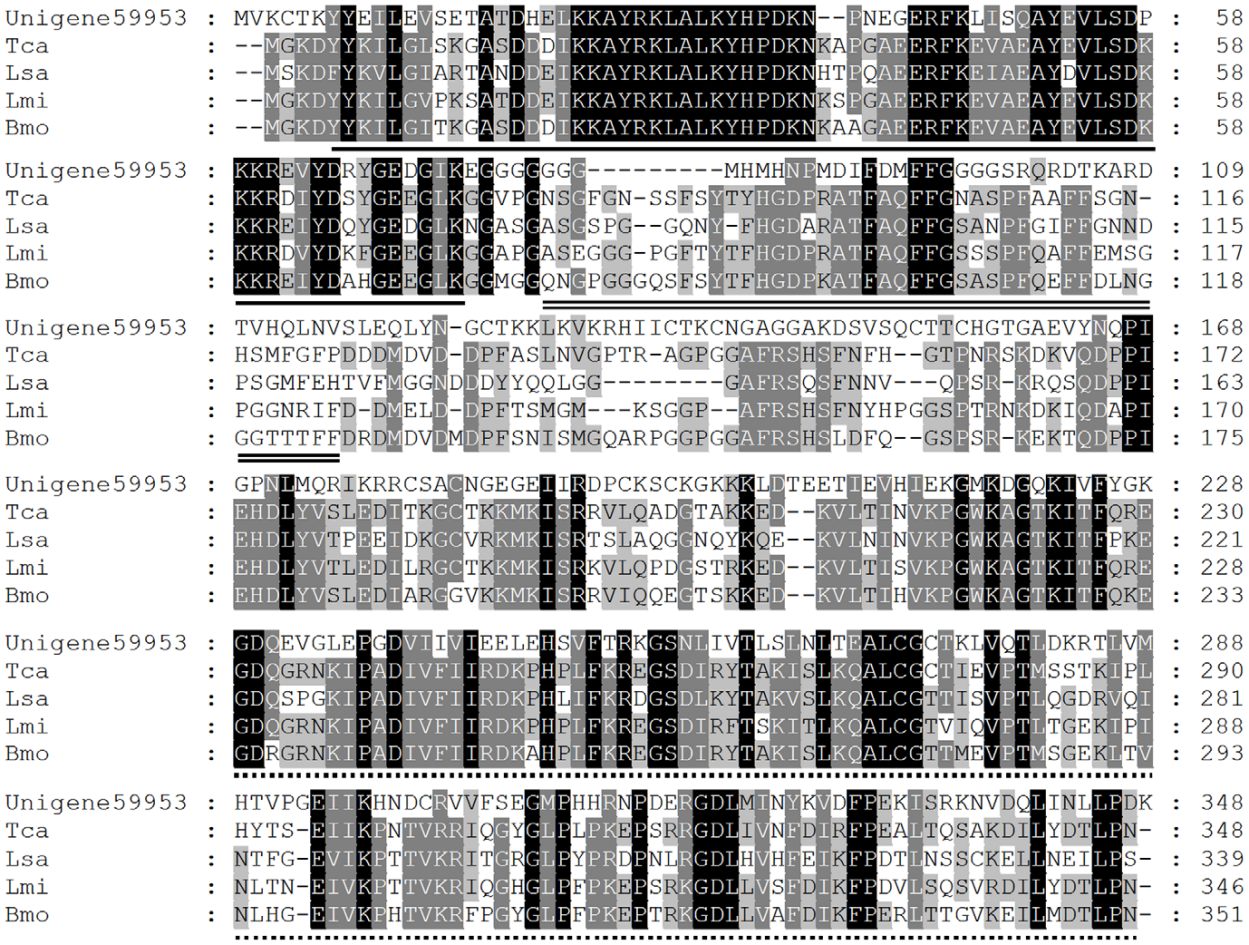
Alignment of the *Tomicus yunnanensis* HSP40 amino acid sequence with other insects HSP40 amino acid sequences. The conserved J domain is underlined. The Gly/Phe-rich domain is double underlined. The cysteine-rich domain is indicated as dotted line. Identical residues are shaded black, conserved substitutions are shaded grey. Dash (–) indicates insertion or deletion. Tca, *Tribolium castaneum* (EFA11191); Lsa, *Liriomyza sativae* (ABE57132); Lmi, *Locusta migratoria* (ABC84495); Bmo, *Bombyx mori* (BAD90846).

HSP40 family is one of the HSPs containing the DNAJ homologous region of *Escherichia coli*, J-domain of DNAJ [42]. HSP40 performs an essential molecular chaperone function in protein translation, folding, unfolding, translocation and degradation, in protein translocation across membranes, and in protecting cells from the effects of heat and other stress factors, primarily acting as cofactors or regulators of heat shock protein 70 (HSP70) [43]. Thirty seven unigenes with similarity to HSP40 were present in *T. yunnanensis* transcriptome. Among them, Unigene59953 is with full open reading frame. The amino acid identity is about 30% compared with those of *T. castaneum*, *Liriomyza sativae*, *Locusta migratoria*, and *Bombyx mori* (Figure 7). Typically, HSP40 proteins have three distinct domains: (1) the J domain of 70 amino acid residues in length which constitutes the most conserved region of these proteins and interacts with HSP70 and stimulates its ATPase activity; (2) a glycine and phenylalanine-rich region (G/F domain), postulated to act as a flexible hinge needed to activate the substrate binding properties of Hsp70 when it interacts with Hsp40; and (3) a cysteine-rich region (C domain) resembling a zinc-finger like structure, suggested to mediate dimer formation and molecular chaperone–peptide interactions [44]–[47]. These three domains were found in the predicted amino acid sequence of *T. yunnanensis* HSP40. However, not all HSP40 necessarily contain all of these 3 domains. Based on the differences in these regions, HSP40 proteins can be categorized into three groups: Type I homologs have all 3 domains (J, G/F, and C), Type II have the J and G/F but not the C domain, and Type III have the J domain alone [48], [49]. According to this, *T. Yunnanensis* HSP40 obtained here belongs to Type I.

#### Heat shock protein 60 (HSP60)

**Figure 8:**
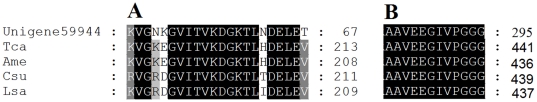
Alignment of the partial amino acid sequence of *Tomicus yunnanensis* HSP60 with that of other insects. (A) the ATP-binding motif. (B) The classical mt-HSP60 signature (underlined) motif. Identical amino acids are shaded black, and conserved residues are shaded grey. Tca, *Tribolium castaneum* (XP_971630), Ame, *Apis mellifera* (XP_392899), Csu, *Chilo suppressalis* (ACT52824); Lsa, *Liriomyza sativae* (AAW49251).

HSP60, a major group of the heat shock proteins, includes stress inducible and constitutively expressed members. It is believed to be predominately mitochondrial, although some are also reported in cytosol and in extracellular compartments [50]. Eighteen unigenes coding for putative HSP60 were identified in the database (Table S5). Compared with orthologues from other organisms, the percentage identity of the deduced amino acid sequence of these unnigenes is high, between 45–93%, confirming the remarkable conservation of this family. Among them, only Unigene59944 was with the length above 1 kb, the others were shorter than 600 bp. Alignment of the predicted amino acid sequence of Unigene59944 with that of other insects revealed it contained the signature peptide known as conserved ATP-binding motif [51] (Figure 8). The classical mitochondrial HSP60 signature motif was found in the deduced amino acid sequence [52], suggesting the HSP60 coded by Unigene59944 presented here is a member of the mitochondrial HSP60 chaperone family. As Unigene59944 was 3′ and 5′-truncted, a fragment of about 30 amino acids at the N terminus required for import into the mitochondriaa and a typical GGM repeat motif for HSP60 at the C terminus were not observed.

#### Heat shock protein 70 (HSP70)

**Figure 9:**
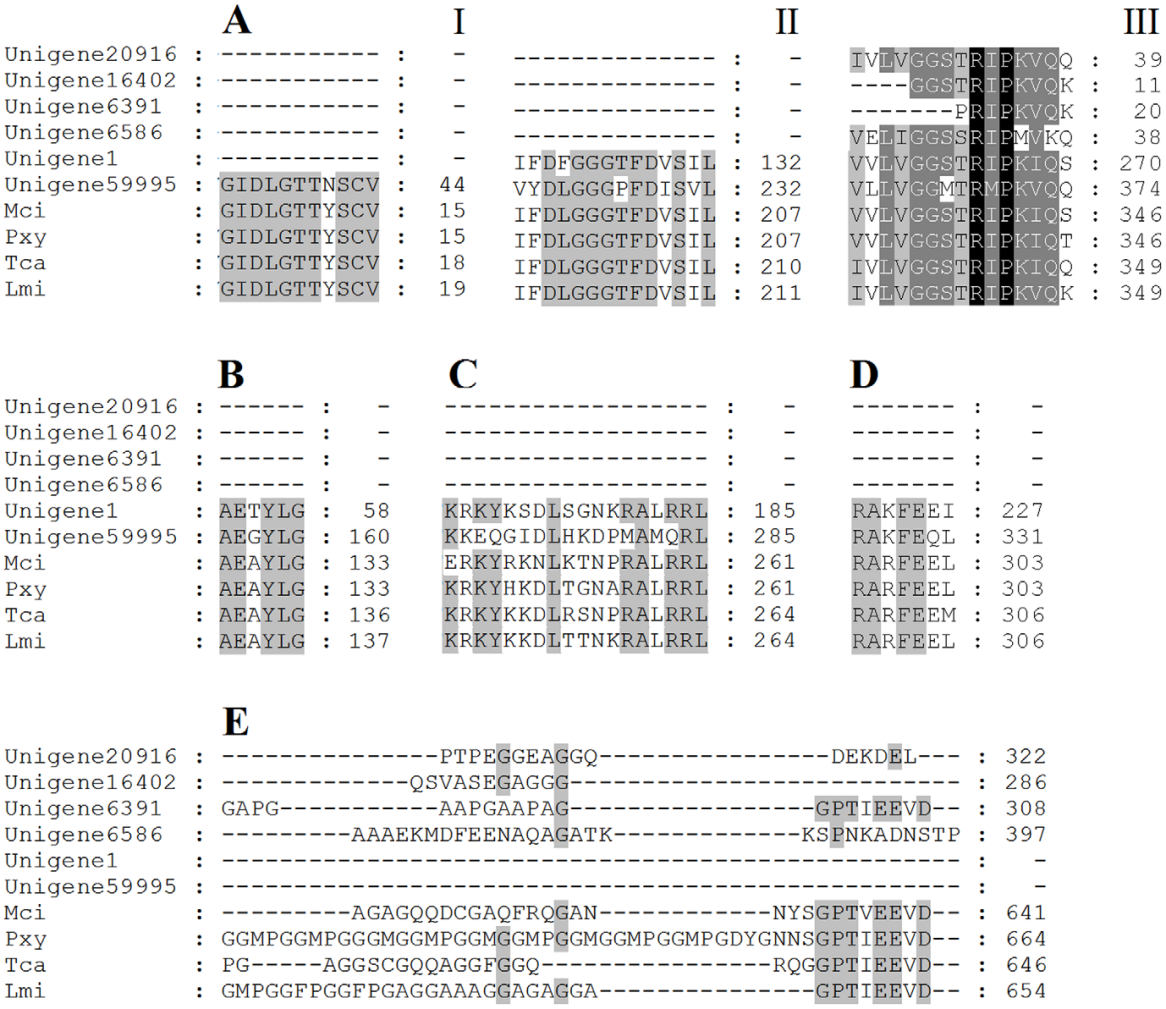
Alignment of the partial amino acid sequence of *Tomicus yunnanensis* HSP70s with that from other insects. (A) Three highly conserved HSP70 family signatures labeled I, II, III. (B) The ATP-GTP binding site. (C) Putative bipartite nuclear localization signals. (D) The nonorganellar consensus motif. (E) The C-terminus. Identical amino acids are shaded black, and conserved residues are shaded grey. Dash (–) indicates insertion or deletion. Mci, *Macrocentrus cingulum* (ACD84944); Pxy, *Plutella xylostella* (BAF95560); Tca, *Tribolium castaneum* (XP_974442); Lmi, *Locusta migratoria* (AAO21473).

In the HSP family, the most studied are HSP70s. There are two main forms of these 70 kD proteins, the heat shock cognate (HSC70) which is expressed constitutively and an inducible form (HSP70) which is normally expressed in response to external stimuli [53]. Seventeen four unigenes produced the best sequence matches to HSP70 (Table S5). The majority of them showed highly conserved identity above 70% to the classic inducible form of HSP70 from other organisms. All these sequences were not in completeness, and only six sequences (Unigene1, Unigene16402, Unigene20916, Unigene59995, Unigene6391, and Unigene6586) were in length longer than 1 kb. After alignment of these 6 non-redundant sequences to that of other insects (Figure 9), three signature motifs of the HSP70 family [54] were conserved. Putative ATP-GTP binding site [55] and nonorganellar consensus motif were located at the amino acid sequences. Putative bipartite nuclear localization signals, which are needed for the selective translocation of HSC70 into the nucleus [56] were identified. The terminal sequence motif of the HSP70 family identifies their cellular location, with the EEDV sequence motif attesting to its cytoplasmic localisation, whereas K/HDEL and PEAEYEEAKK characterise members localised to the endoplasmic reticulum and mitochondria respectively [53], [57]. With regard to this, the terminal sequence motif of Unigene20916 and Unigene6391 were K/HDEL and EEDV, indicating they belonged to cytoplasmic localization and endoplasmic reticulum member, respectively.

#### Heat shock protein 90 (HSP90)

In normal physiological conditions, HSP90 is abundant, accounting for 1% of the total soluble cytosolic protein, which is a highly conserved molecular chaperone contributing to the folding, maintenance of structural integrity and proper regulation of a subset of cytosolic protein [54], [58], [59]. Like HSP70, HSP90s has also been extensively studied. Twenty four unigenes with short length less than 800 bp were identified coding for putative HSP90 (Table S5). Except for Unigene20269 and Unigene46492, they showed above 60% amino acid identity to HSP90s from other organisms. Other sequence information was not analyzed here because of only relatively short fragments available.

### Conclusions

In this work, transcriptome database has been produced on a large scale for *T. yunnanensis* using Illumina sequencing. The results provided new insights into the genomics of this forest pest. It contributed significantly to the rapid discovery of a wide diversity candidate gene for this organism that lacks complete genome sequences and other genetic tools and resources. This transcriptome can be used as a reference to provide new leads for comparative studies within the family. Based on this database, the predicted repertoire of genes related to insecticide resistance and environmental stress are constructed, providing valuable information regarding further investigations of the detailed mechanisms. However, as the sequences obtained by Illumina sequencing are short, most of the interesting putative genes discovery by this comprehensive tool is need to rely on RACE PCR in order to obtain full-length sequence data. Furthermore, the database will continue to be an enormous resource for genome-wide association studies of the whole picture of other important biological questions in the future.

## Supporting Information

Figure S1 Length distribution of contigs.
(TIF)

Figure S2 Length distribution of scaffolds.
(TIF)

Table S1 Top BLAST hits from NCBI nr database. BLAST results against the NCBI nr database for all the distinct sequences with a cut-off E value above 10^−5^ are shown.
(XLS)

Table S2 Gene Ontology of *Tomicus yunnanensis* unigenes.
(XLS)

Table S3 KEGG summary of *Tomicus yunnanensis* transciptome.
(XLS)

Table S4 Putative P450 genes identified in *Tomicus yunnanensis* transcriptome.
(XLS)

Table S5 Putative heat shock protein genes identified in *Tomicus yunnanensis* transcriptome.
(XLS)
