# Supplementary figures and images for: Global Transcriptome Profiling of the Pine Shoot Beetle, Tomicus yunnanensis (Coleoptera: Scolytinae)
Source: PLoS One. 2012 Feb 23;7(2):e32291. doi: 10.1371/journal.pone.0032291 (PMC3285671; doi:10.1371/journal.pone.0032291)

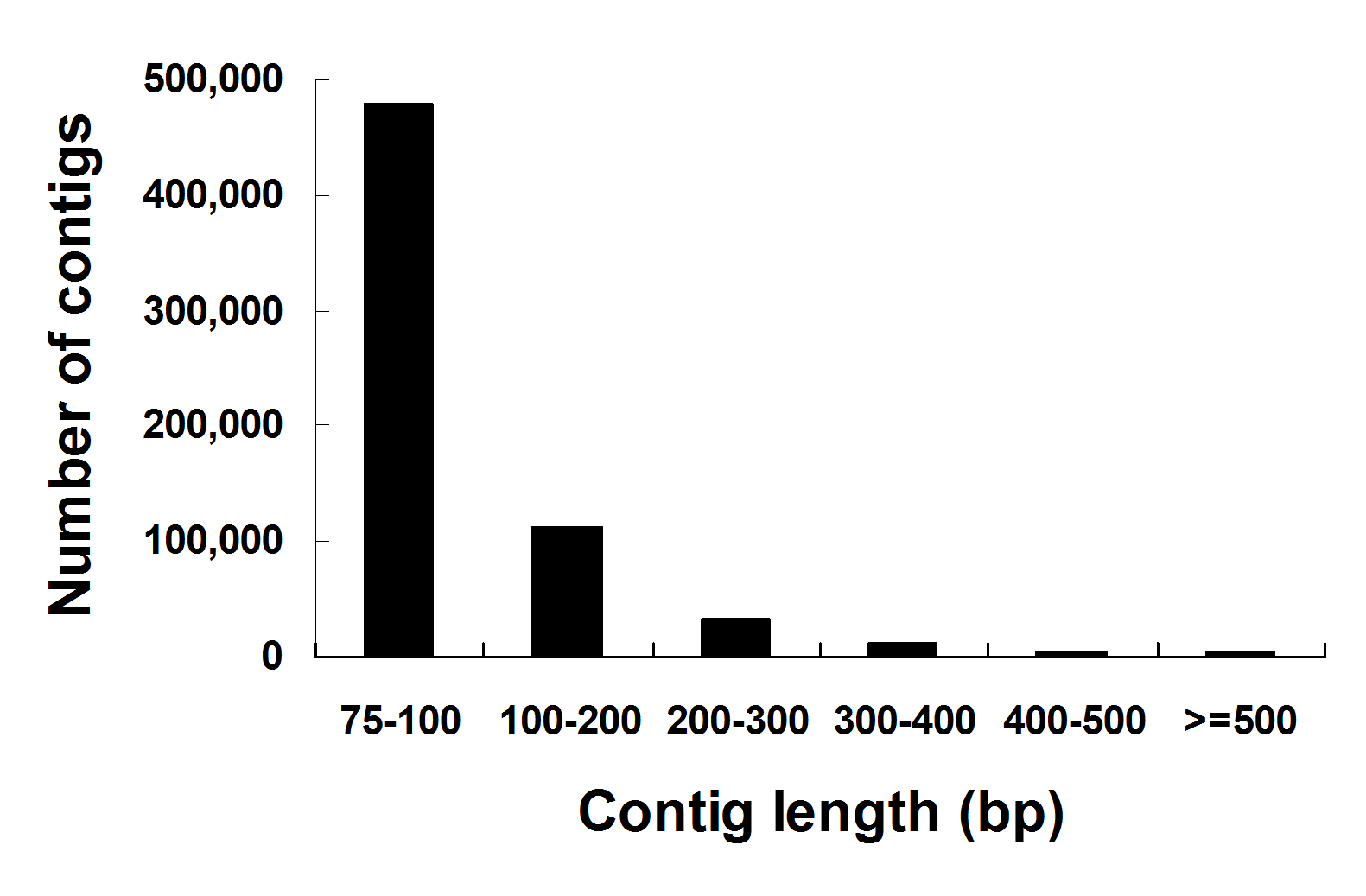

Supplement: Figure S1 — Length distribution of contigs. (TIF) [file pone.0032291.s001.tif]

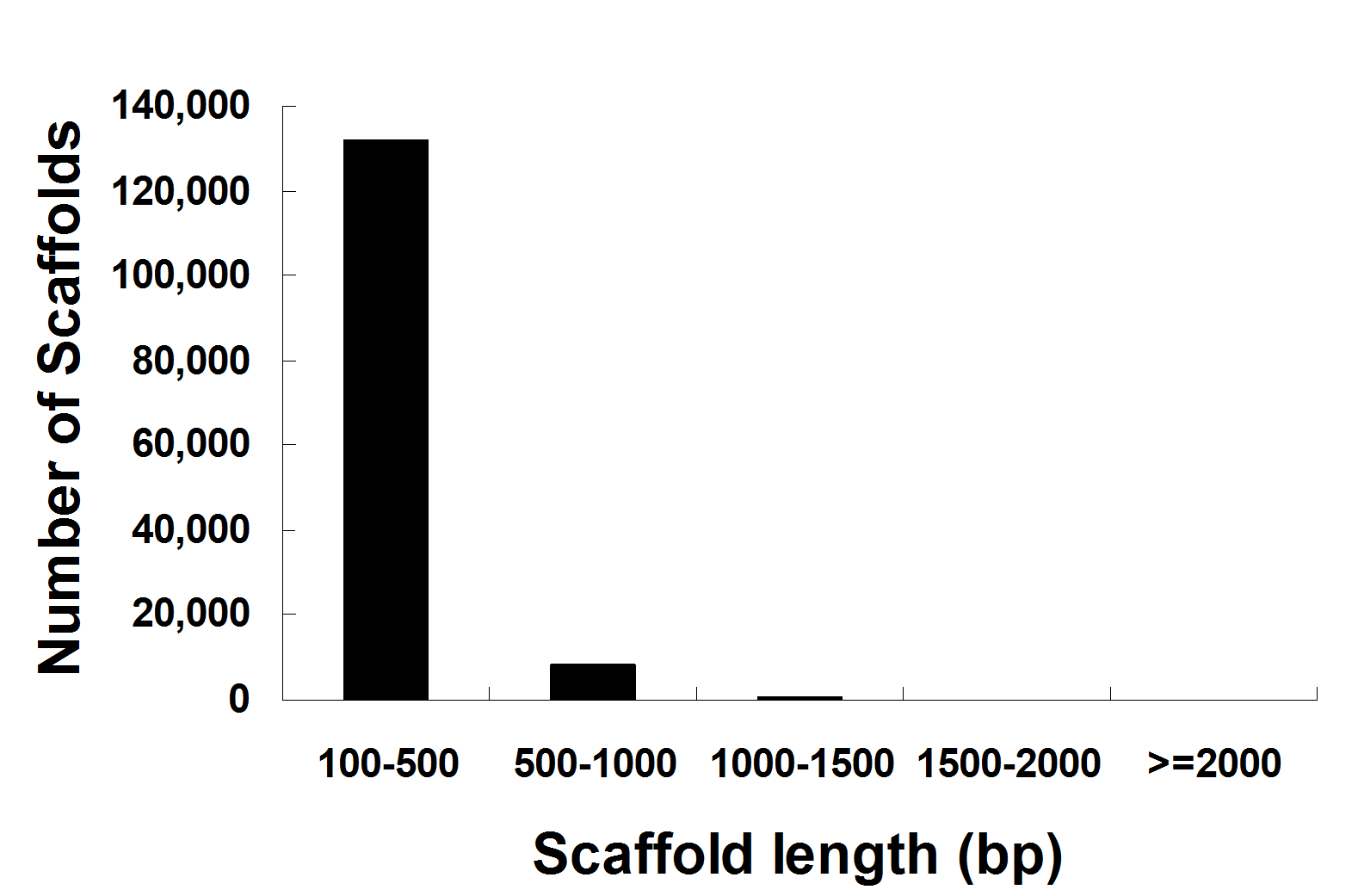

Supplement: Figure S2 — Length distribution of scaffolds. (TIF) [file pone.0032291.s002.tif]
